# Radical Scavenging Capability and Mechanism of Three Isoflavonoids Extracted from Radix Astragali: A Theoretical Study

**DOI:** 10.3390/molecules28135039

**Published:** 2023-06-28

**Authors:** Xiao-Qin Lu, Shu Qin, Jindong Li

**Affiliations:** Shanxi Center for Testing of Functional Agro-Products, Shanxi Agricultural University, Taiyuan 030031, China; luxiaoqin@sxau.edu.cn (X.-Q.L.); qinshu55@126.com (S.Q.)

**Keywords:** antioxidant mechanism, radical scavenging reaction, isoflavonoids, DFT, structure-activity relationship

## Abstract

As a valuable traditional Chinese herbal medicine, Radix Astragali has attracted much attention due to its extensive pharmacological activities. In this study, density functional theory (DFT) was used thermodynamically and kinetically in detail to predict the antioxidant activity and reaction mechanisms involved in the free radical scavenging reactions of three representative isoflavonoids (formononetin, calycosin, and calycosin-7-glucoside) extracted from Radix Astragali. Three main mechanisms, including hydrogen atom transfer (HAT), proton transfer after electron transfer (SET-PT), and sequential proton loss electron transfer (SPLET) were examined by calculating the thermodynamic parameters. It was found that HAT is the predominant mechanism in the gas phase, while SPLET is supported in the solvent environment. The isoflavonoids’ order of antioxidant activity was estimated as: calycosin > calycosin-7-glucoside > formononetin. For the calycosin compound, the result revealed the feasibility of double HAT mechanisms, which involve the formation of stable benzodioxazole with significantly reduced energy in the second H^+^/e^−^ reaction. In addition, the potential energy profiles and kinetic calculations show that the reaction of ^•^OH into the 3′-OH site of calycosin has a lower energy barrier (7.2 kcal/mol) and higher rate constant (4.55 × 10^9^ M^−1^ s^−1^) compared with other reactions in the gas phase.

## 1. Introduction

Reactive oxygen species (ROS) are generated by normal metabolic processes and external stimuli within the cell, and play a key role in regulating many physiological functions. However, inappropriate scavenging or an excessive build-up of ROS in the body would lead to oxidative stress, which usually results in tissue damage and is eventually implicated in aging and a variety of diseases, such as cancer as well as atherosclerosis and neurodegenerative disorders [1,2,3,4]. The most promising strategy for defending against oxidative damage is the usage of antioxidants or radical scavengers [5]. Therefore, searching and identifying efficient antioxidant compounds has become an increasingly important area of research.

**Figure 1:**
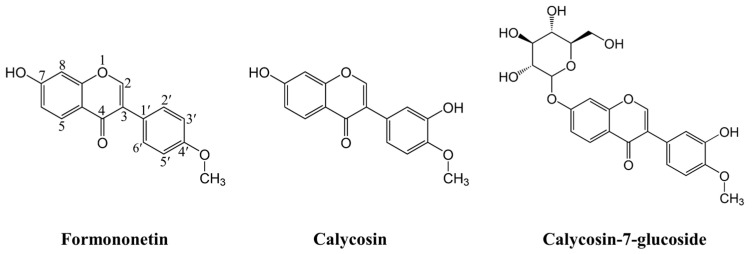
Chemical structures and atom numbering sites of three isoflavonoid components extracted from Radix Astragali.

Radix Astragali, one of the most popular traditional Chinese herbal medicines, has been widely used in health foods and dietary supplements to enhance immunity and treat diseases for thousand years in China [6,7]. Several studies have indicated that Radix Astragali has various pharmacological functions, including antitumor [8], antioxidation [9], anti-inflammation [10], and neuroprotection [11], which are highly correlated with its bioactive components. Isoflavonoids, as a subclass of flavonoids, including formononetin, calycosin, and its glucoside, named calycosin-7-glucoside (Figure 1), are important bioactive constituents of Radix Astragali and contribute to multiple biological activities of Radix Astragali [12]. They are often used as standard components for the quality evaluation of Radix Astragali. In recent years, many advanced technologies in experiments have been adopted to extract and separate bioactive components from Radix Astragali [13,14]. Interestingly, formononetin, calycosin, and calycosin-7 glucoside were found to be active against free radicals as evaluated by DPPH radical scavenging activity and oxygen radical absorbance capacity (ORAC) assays, exhibiting excellent antioxidant activity [15,16,17,18].

Experimental studies on the antioxidant behavior of compounds have attracted widespread attention from theoretical researchers. With the development of theoretical approaches, theoretical calculations have been used in multiple research fields [19,20,21,22]. Many investigations have demonstrated the successes of different density functional theory (DFT) methods in revealing structure–activity relationships, determining the most likely chemical reactions involved in the antioxidant activity, as well as explaining the experimental results [23,24,25,26,27,28,29]. Compared with the experimental studies, theoretical calculations based on DFT can be considered as cogent tools in elucidating the antioxidant activity of flavonoid compounds with lower cost and shorter time requirements. It is well known that the free radical scavenging capacity of flavonoids is largely affected by the number and position of hydroxyl groups in their molecular structures. The reaction mechanisms involved in free radical scavenging by hydroxyl groups have been found to mainly include hydrogen atom transfer (HAT), single electron transfer followed by proton transfer (SET-PT), and sequential proton loss electron transfer (SPLET) [30,31,32]. Additionally, the double H^+^/e^−^ free radical scavenging mechanism has also been identified. For example, Amić et al. pointed out that cinnamic acid derivatives may scavenge free radicals via a double H^+^/e^−^ reaction through the participation of a catechol or guaiacyl moiety [33]. At the same time, studies on the contributions of substituents to the radical scavenging capability have also been reported. Introducing an electron-donating group (−NH_2_) into genistein has been found to effectively improve its antioxidant activity [34]. Zheng et al. demonstrated that introducing glycoside groups at different hydroxyl sites of quercetin can have different effects on its antioxidant activity [35].

In this work, we attempted to theoretically evaluate the antioxidant activity of the formononetin, calycosin, and calycosin-7-glucoside extracted from Radix Astragali by performing systematic DFT studies. The main goal of this research is to shed light on the structure–radical scavenging activity relationship and the possible multiple mechanisms underlying the radical trapping process. The thermodynamic parameters involved in the three well-established radical scavenging mechanisms were calculated in the gas phase and in solution, including the bond dissociation enthalpy (BDE), ionization potential (IP), proton dissociation enthalpy (PDE), proton affinity (PA), and electron transfer enthalpy (ETE). Additionally, calycosin bearing a guaiacyl moiety proceeds with a double free radical trapping reaction. Furthermore, the potential energy surfaces (PES) and rate constants (*k*) of the reaction between the calycosin and radical ^•^OH/^•^OCH_3_ were explored for an insight into their mechanism of action. The frontier molecular orbital and natural bond orbital (NBO) analyses were executed to evaluate the free radical scavenging ability of the abovementioned isoflavonoid components.

## 2. Results and Discussion

### 2.1. Conformational Analysis

The conformational structures of flavonoids are closely related to their ability to resist the attacks of free radicals. In order to select the predominant conformer for further studies, we first performed the conformational search for formononetin, calycosin, and calycosin-7-glucoside using the Molclus program [36]. Low-lying isomers were subsequently optimized at the M06-2X level [37] with the 6-311 + G(d,p) basis set [38] using the Gaussian 16 package [39], with the vibrational frequencies checked. The most stable optimized conformer of the three isoflavonoid compounds in the gas phase are depicted in Figure S1. As can be seen, all of the studied molecules adopt nonplanar geometrical structures. A comparison of formononetin with other studied isoflavonoids showed that the introduction of substituents (hydroxyl group or glycosyl group) in the B-or A-ring only causes slight geometrical changes. Starting from the structures of the most stable conformer, the structures of the corresponding radicals, radical cations, and anions of the studied isoflavonoids were optimized at the same level. No significant geometrical changes were observed when abstracting the hydrogen atom from each phenolic OH group for all studied isoflavonoids.

### 2.2. Analysis of Free Radical Scavenging Reaction Paths

To predict the free radical scavenging activity of formononetin, calycosin, and calycosin-7-glucoside, the thermodynamic parameters involved in the free radical scavenging mechanisms were calculated. The first H^+^/e^−^ reaction may proceed via the HAT, SET-PT, or SPLET mechanisms. For calycosin with more than one hydroxyl group, it is found that the phenoxyl radicals generated in the first H^+^/e^−^ reaction may proceed in a second H^+^/e^−^ reaction to trap free radicals.

#### 2.2.1. HAT Mechanism

In the HAT mechanism, the BDE is usually regarded as a reliable thermochemical parameter to evaluate the radical scavenging activity of antioxidants. A lower BDE indicates a higher radical scavenging capacity of the investigated compounds. The BDEs of formononetin, calycosin, and calycosin-7-glucoside computed at the M06-2X/6-311 + G(d,p) level in the gas, water, and ethanol phases are listed in Table 1. Ethanol is the common organic solvent used for extraction, which is non-polluting, non-toxic, and cost-effective. The solvent effects were investigated using the SMD continuum solvation model [40]. For formononetin with only one hydroxyl group in ring A (7-OH), the BDEs were calculated to be 108.3, 95.6, and 94.4 kcal/mol in the three media, respectively. When another hydroxyl group was introduced into ring B (calycosin), the BDEs of 7-OH maintained the same value in the gas phase, whereas they increased by 8.6 and 10.5 kcal/mol in the water and ethanol phases, respectively. Moreover, the BDEs of 3′-OH in calycosin were calculated to be 87.1, 85.1, and 84.3 kcal/mol in the three media, respectively, which are always lower than those of 7-OH calculated at the same level, indicating that 3′-OH should determine the H-donating ability of calycosin. For the calycosin-7-glucoside compound, it can be seen as replacing the 7-OH of the A-ring of calycosin with a glycosyl group. Surprisingly, the BDEs of 3′-OH were calculated to be 87.3, 85.1, and 84.5 kcal/mol in the three media, respectively, indicating that the presence of a glycosyl group at the 7-OH site of the A ring of the calycosin-7-glucoside compound has almost no influence on the 3’-OH in the B ring. As revealed in Table 1, the solvent effects only exert a slight influence on the BDEs of the isoflavonoids because no charged species are involved in the HAT process.

In addition, the natural bond orbital (NBO) analyses [41] were also performed on the hydrogen atoms of phenolic hydroxyls for formononetin, calycosin, and calycosin-7-glucoside. As listed in Table S1, the results showed that the charge on the hydrogen atom of 3’-OH was higher than that of 7-OH, indicating that 3’-OH had higher activity and was more prone to reacting with oxygen radicals. Based on the above results, the hydrogen-donating ability of the isoflavonoids obeys the order of calycosin > calycosin-7-glucoside > formononetin.

#### 2.2.2. SET-PT Mechanism

Apart from the HAT mechanism, SET-PT is another significant mechanism for flavonoid compounds to scavenge free radicals. The calculated IP and PDE parameters involved in the SET-PT pathway for the studied isoflavonoids in different media are summarized in Table 1. The first step of the SET-PT mechanism is electron transfer, which is determined by the IP. It can be found that in the gas phase, the IPs of formononetin, calycosin, and calycosin-7-glucoside obey the order of calycosin (172.9 kcal/mol) < calycosin-7-glucoside (173.0 kcal/mol) < formononetin (175.2 kcal/mol). Moreover, an analogue tendency is also found in water and ethanol solvent, whereby calycosin < calycosin-7-glucoside < formononetin. These results indicate that calycosin is more prone to donate electrons than the others in all three media, whereas formononetin is the least effective one. In addition, compared to the BDEs, solvents have a significant impact on the IP values. The IPs decreased by ~43 and 49 kcal/mol in water and ethanol solvents, respectively, in comparison to those in the gas phase. This means that the polar solvents favor the electron abstraction from the studied isoflavonoids.

The PDE is the parameter for the second step in the SET-PT mechanism, which involves deprotonation from the radical cation yielded in the first step. It was found that the calculated PDEs for the investigated compounds dramatically decreased by averages of 221 and 224 kcal/mol from the gas phase to the water and ethanol phases, respectively, due to the high solvation enthalpy of protons in solution. The PDE of 3′-OH is smaller than the PDE of 7-OH, which means that the second step of the SET-PT mechanism of calycosin and calycosin-7-glucoside is favored by 3′-OH to donate protons from its radical cation.

By comparison, it can be found that in three studied media, the BDE values are always lower than the IP values. This indicates that the HAT mechanism is a more preferable process for trapping radicals than the SET-PT mechanism.

#### 2.2.3. SPLET Mechanism

The SPLET mechanism consists of the deprotonation of the phenolic OH group, followed by electron transfer from the phenoxyl anion. The two descriptors of PA and ETE were used to explore the probability of the SPLET mechanism for the investigated compounds. Similar to the SET-PT mechanism, the first step of the SPLET mechanism also plays an important role, with the lowest IP and PA values indicating the main mechanism and reaction pathway from a thermodynamic perspective. The obtained results are given in Table 1. The order of the PAs of the investigated isoflavonoids in the three media is gas >> ethanol > water. As an example, the PAs of 3′-OH in calycosin decrease from 348.4 kcal/mol in the gas phase to 38.7 and 36.4 kcal/mol in water and ethanol, which indicates that deprotonation occurs more easily in the solvents. For the investigated compounds, the PAs of 3′-OH are slightly bigger than that of 7-OH. This result indicates that the formation of the isoflavonoid-O7^−^ anion is easier than the isoflavonoid-O3′^−^ anion in the studied environments.

By comparison, it can be clearly observed that the PAs in the solvent phase are significantly lower than the corresponding BDEs and IPs, indicating that the SPLET mechanism dominates the reaction pathway in the solvent phase. The ETEs are higher in the solution environment and lowest in the gas phase, which is caused by the larger solvation enthalpy of the anion rather than the electron and neutral radical. These results are in line with previous reports [42,43]. In addition, when compared to the PAs and ETEs of the same O-H site in the different investigated compounds, the values do not change much. For example, it is found that the substitution of the 7-OH group with glycosyl group has little influence on the acid strength of the 3′-OH of the calycosin-7-glucoside compared with calycosin and the donating electron ability of the corresponding anions.

#### 2.2.4. Double HAT Mechanism

**Figure 2:**
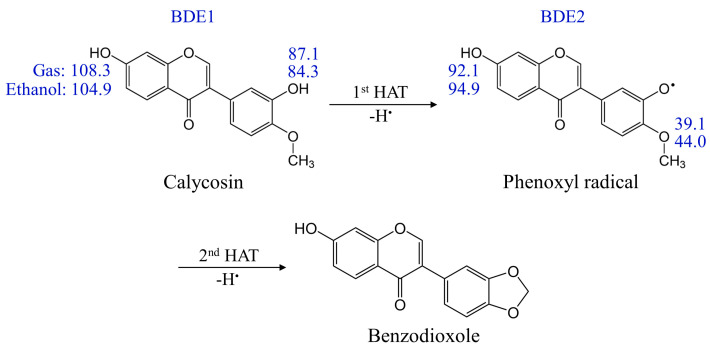
Double HAT mechanism of calycosin in the gas phase and ethanol solvent (unit: kcal/mol).

Here, we further explored the thermodynamic feasibility of the double HAT (dHAT) mechanism for the calycosin. To better illustrate the process, the dHAT mechanism in the gas and ethanol phases for the calycosin is given in Figure 2. The results indicate that the phenoxyl radical can generate stable benzodioxole (singlet state) through cyclization using twisted ortho-OCH_3_ groups rather than the phenoxyl diradical (triple state) after the second H^+^/e^−^ reaction. Thus, the OCH_3_ group is the preferred site for the second HAT. As can be seen, the BDEs for the formation of benzodioxazole are only 39.1 and 44.0 kcal/mol in the gas phase and ethanol, respectively, which are ~53 and 51 kcal/mol more stable than the formation of the phenoxyl diradical, respectively, similar to previous studies [24,27]. These values are lower than the corresponding BDEs of the 3′-OH and 7-OH in the first HAT, which suggests the high antioxidant capacity of the generated phenoxyl radicals and the feasibility of the second HAT process from the OCH_3_ group for trapping free radicals.

### 2.3. Kinetic Study

**Figure 3:**
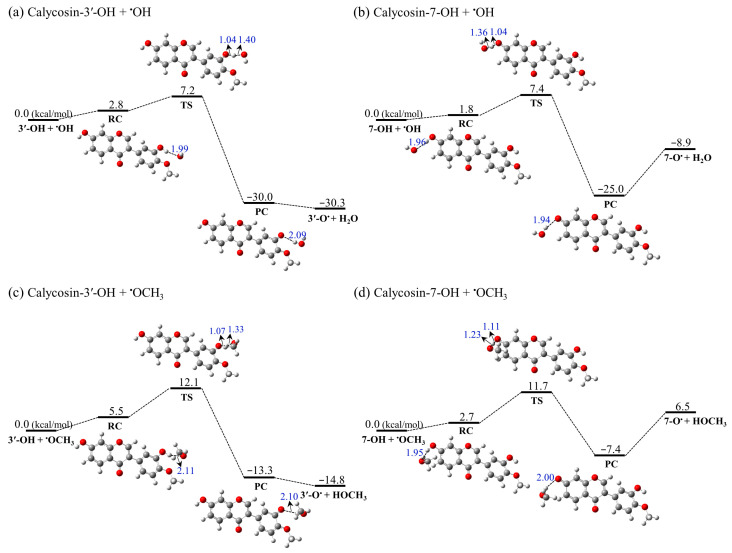
PESs of the HAT reaction between the O3′ and O7 positions of calycosin and the ^•^OH/^•^OCH_3_ radical in the gas phase. RC, TS, and PC represent the pre-complex, transition state, and post-complex, respectively. The reaction Gibbs free energies (ΔG) are indicated in kcal/mol at the M06-2X/6-311 + G(d,p) level. The distances of O···H are highlighted in blue (unit: Å). The elements C, O, and H are indicated in gray, red, and white, respectively.

Apart from the thermodynamic approach, in order to obtain further insights into the free radical scavenging activity of the antioxidants, we performed a systematic study on the reaction pathway of calycosin with different ROS such as hydroxyl (^•^OH) and methoxy (^•^OCH_3_) radicals via the HAT mechanism. Table 2 summarize the reaction kinetics and thermodynamics of the O3′ and O7 positions of calycosin with ^•^OH/^•^OCH_3_ radicals. Here, to better understand the H donation process, the potential energy surfaces (PES) were calculated for the reactions between ^•^OH/^•^OCH_3_ and the different sites of calycosin, correspondingly (Figure 3). The rate constants were also calculated using the KiSThelP 2019 program [44], which are useful criteria for identifying the most efficient compounds for scavenging radicals. All calculations were performed in the gas phase at the M06-2X/6-311 + G(d,p) level.

#### 2.3.1. Reaction with ^•^OH

As one of the most reactive oxygen-centered free radicals, ^•^OH usually reacts with almost all biological molecules in its vicinity and causes oxidative damage to tissues. As can be seen in Figure 3a,b and Table 2, it was found that the reactions of the O3′ and O7 positions of calycosin with the ^•^OH radical are always exergonic relative to the reactant complexes, with the Gibbs free energy values falling to 30.3 and 8.9 kcal/mol in the gas, respectively. The average activation Gibbs free energy (ΔG^≠^) value for calycosin is 7.3 kcal/mol, indicating shallow barriers when the H atom transfer reacts with ^•^OH. This result is in keeping with other findings for polyphenol [28,45]. A comparison of the Gibbs free energy values and barriers of the two positions of calycosin showed that 3′-OH is the more active site for radical attacks, which is consistent with the results obtained in the thermodynamical calculations. Examining the optimized structures of TSs, the distances of breaking O···H bonds are 1.04 and 1.04 Å, whereas the forming H···O bonds are 1.40 and 1.36 Å, respectively, indicating the existence of an early transition state. Furthermore, the rate constant of the calycosin-3′-OH + ^•^OH reaction is 4.55 × 10^9^ M^−1^ s^−1^, which is higher than that of the calycosin-7-OH + ^•^OH reaction (2.03 × 10^9^ M^−1^ s^−1^).

#### 2.3.2. Reaction with ^•^OCH_3_

Methoxy radicals (^•^OCH_3_), as the simplest members of the alkoxy group family (RO^•^), have moderate reactivity compared to the high reactivity of ^•^OH radicals. The Gibbs free energies of the overall reaction (ΔG) and activation barriers (ΔG^≠^) associated with the radical abstraction channels of the calycosin molecule against ^•^OCH_3_ in the gas phase are provided in Figure 3c,d and Table 2. The reaction energy barriers of the O3′ and O7 positions of calycosin with the ^•^OCH_3_ following the HAT mechanism are 12.1 and 11.7 kcal/mol, respectively, which are considerably higher than those of ^•^OH. For the 3′-OH of calycosin, the PES tendency of the reaction with ^•^OCH_3_ is quite similar to that of the reaction with ^•^OH. However, it is worth noting that the reaction between the 7-OH of calycosin and ^•^OCH_3_ is endothermic. The rate constant of the calycosin-3′-OH + ^•^OCH_3_ (6.72 × 10^5^ M^−1^ s^−1^) is smaller than that of the ^•^OH radical, which may be attributed to ^•^OH being a stronger electron acceptor than ^•^OCH_3_, meaning it can quickly react with the calycosin molecule through the HAT pathway.

### 2.4. Molecular Orbital Analysis

**Figure 4:**
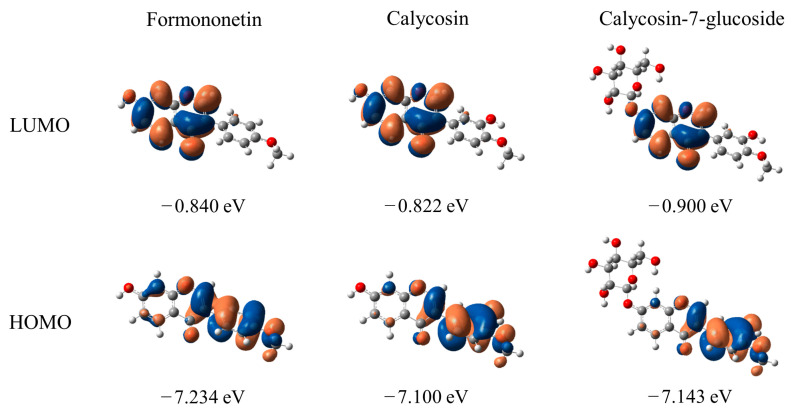
The electron density distribution and energies of the HOMO and LUMO for formononetin, calycosin, and calycosin-7-glucoside calculated at the M06-2X level in the gas phase.

The electron density distribution and energies of the HOMO and LUMO in the gas phase for the studied isoflavonoids are given in Figure 4. The energies of the HOMO and LUMO can been regarded as important parameters in evaluating electron-donating and electron-receiving abilities, respectively. It can be observed that the HOMO energies follow the order of calycosin (−7.100 eV) > calycosin-7-glucoside (−7.143 eV) > formononetin (−7.234 eV), indicating that calycosin would exhibit the strongest electron-donating ability. As shown in Figure 4, the HOMOs of formononetin, calycosin, and calycosin-7-glucoside present a similar electron density distribution, which is mainly localized on the B- and C-rings as well as the 3′-OH group. In contrast, the LUMOs of all compounds are centered on the A- and C-rings. Interestingly, there is no contribution of the glycoside substituent for the HOMO and LUMO of calycosin-7-glucoside, which indicates that H atoms on the glycoside substituent do not easily participate in the reaction.

## 3. Computational Details

The stable conformation of the formononetin, calycosin, and calycosin-7-glucoside were searched by the Molclus program in this study [36]. Further optimization and a frequency analysis for these investigated molecules and their radicals, radical cations, and anions were performed at the M06-2X/6-311 + G(d,p) level of the theory using the Gaussian 16 package [37,38,39]. The M06-2X function is highly recommended for thermodynamic and kinetic calculations. In particular, this functional has been used in many studies for modelling reaction energetics involving free radicals [28,33,46,47]. The influence of water and ethanol as solvents was calculated using the SMD continuum solvation model [40], which has been successfully used for the study of free radical scavenging mechanisms in conjunction with the hybrid M06-2X functional [48]. Unrestricted calculations were used for the radicals. Natural bonding orbital (NBO) analyses were performed using the NBO 6.0 program in order to analyze the distribution of the unpaired electron in the radical species [41].

The transition states, pre-complex (RC), post-complex (PC), and products of the reaction between the ^•^OH/^•^OCH_3_ radicals and calycosin molecules were optimized and calculated at the same level. The RCs, PCs, and products were verified by a normal-mode analysis to be local minima, and the transition states for each reaction were confirmed by having a single imaginary frequency on the potential energy surface. The intrinsic reaction coordinate (IRC) calculations were performed to ensure that the obtained structures were the true TS [49]. All reaction enthalpies and Gibbs free energies were calculated at 298.15 K.

### 3.1. Antioxidant Mechanisms and Thermochemical Parameters

Three common antioxidant mechanisms (HAT, SET-PT, and SPLET) were considered in the free radical scavenging activity of flavonoids (ArOH) [30,31,32]. The thermochemical parameters related to the three antioxidant mechanisms were systematically calculated.

(a) HAT is a one-step mechanism in which hydrogen atoms are transferred from flavonoid hydroxyl groups to the free radicals through homolytic cleavage of the O-H bond (Equation (1)). The activity of the antioxidants can be characterized by the BDE for this mechanism (Equation (2)):

ArOH + R^•^ → ArO^•^ + RH (1)

BDE = H(ArO^•^) + H(H^•^) − H(ArOH) (2)

(b) The SET-PT mechanism consists of two-steps. In the SET-PT mechanism, electron transfer from ArOH is followed by proton transfer (Equation (3)). The first and second step of the SET-PT mechanism are governed by IP and PDE, respectively (Equations (4) and (5)):

ArOH + R^•^ → ArO^•+^ + R^−^ → ArO^•^ + RH (3)

IP = H(ArOH^•+^) + H(e^−^) − H(ArOH) (4)

PDE = H(ArO^•^) + H(H^+^) − H(ArOH^•+^) (5)

(c) For the SPLET mechanism, it also consists of two-steps. Proton transfer from ArOH is followed by electron transfer (Equation (6)). The PA and ETE were used to drive the first and second steps, respectively (Equations (7) and (8)):

ArOH → ArO^−^ + H^+^ + R^•^ → ArO^•^ + RH (6)

PA = H(ArO^−^) + H(H^+^) − H(ArOH) (7)

ETE = H(ArO^•^) + H(e^−^) − H(ArO^−^) (8)

where ArO^•^, ArOH^•+^, and ArO^−^ represent the radical, radical cation, and anion of the flavonoids, respectively. The enthalpy values of the hydrogen atom (H^•^), proton (H^+^), and electron (e^−^) were obtained from the literature [50,51]. The proton and electron solvation enthalpies were computed according to the reported literature [52]. The BDE, IP, and PA values were used as the main thermodynamic parameters to explain the radical scavenging activity of the studied compounds.

### 3.2. Kinetic Parameters

The rate constants were calculated using the KiSThelP 2019 program [44] at 1 M standard state in the gas phase. Based on conventional transition state theory (TST) [53], the rate constants were calculated as follows:

(9) $$k_{TST}=\sigma_{k}\frac{k_{B}T}{h}\exp\left(-\frac{\Delta G^{\#}}{\mathrm{RT}}\right)\;$$

where *k*_B_ is the Boltzmann constant, *T* is the temperature, *h* is the Planck constant, Δ*G*^#^ is the Gibbs free energy of activation, *σ* is the reaction path degeneracy, and *k* accounts for tunneling corrections, which are calculated through the Wigner approaches [54]. 

## 4. Conclusions

In this paper, the antioxidant properties of three isoflavonoid components (formononetin, calycosin, and calycosin-7-glucoside) extracted from Radix Astragali were studied using the DFT method through the HAT, SET-PT, and SPLET mechanisms. The thermodynamic descriptors including the BDE, IE, PDE, PA, and ETE were calculated in the gas, water, and ethanol phases for the radical scavenging activity. The conclusions are summarized as follows:

(1) The hydroxyl group on the O3′ position has a higher H-atom donation ability than that on the O7 position for the investigated compounds. A comparison of the intrinsic thermodynamic properties including BDEs, IPs, and PAs demonstrated that the HAT action is thermodynamically preferred in the gas phase and SPLET is more preferred in the solvent phase in the first H^+^/e^−^ reaction. The sequence of free radical scavenging capability for the three isoflavonoid compounds is calycosin > calycosin-7-glucoside > formononetin.
(2) The calycosin preferentially undergoes the first H^+^/e^−^ reaction on the 3′-OH site, followed by the second H^+^/e^−^ reaction from the ortho-OCH_3_ group to form stable benzodioxazole with considerably reduced energy via the double HAT mechanism.
(3) The potential energy profiles and kinetic calculations show that the reaction of ^•^OH into the 3′-OH site of calycosin has a lower energy barrier (7.2 kcal/mol) and higher rate constant (4.55 × 10^9^ M^−1^ s^−1^) compared with other reactions. It is worth noting that the reaction between the 7-OH of calycosin and ^•^OCH_3_ is endothermic.

These results contribute to a deeper understanding of the antioxidant activity of the Radix Astragali. We believe that our findings will provide a theoretical basis for the development and application of natural antioxidants.

## Figures and Tables

**Table 1:** Relative enthalpy energies (BDE, IP, PDE, PA, and ETE, in kcal/mol) of the three major antioxidant mechanisms for the studied compounds calculated at the M06-2X/6-311 + G(d,p) level in the gas phase and solvent.

Mechanism	HAT			SET-PT						SPLET					
	BDE			IP			PDE			PA			ETE		
Compounds	Gas	Water	Ethanol	Gas	Water	Ethanol	Gas	Water	Ethanol	Gas	Water	Ethanol	Gas	Water	Ethanol
**Formononetin**				175.2	132.9	127.2									
7-OH	108.3	95.6	94.4				246.5	16.7	14.3	328.9	30.7	31.7	92.8	118.9	109.8
**Calycosin**				172.9	129.3	123.7									
3′-OH	87.1	85.1	84.3				227.6	9.7	7.6	348.4	36.4	38.7	52.1	102.6	92.7
7-OH	108.3	104.2	104.9				248.7	28.8	28.2	329.2	30.7	31.7	92.5	127.4	120.3
**Calycosin-7-glucoside**				173.0	129.7	124.1									
3′-OH	87.3	85.1	84.5				227.7	9.4	7.4	347.3	36.4	38.7	53.3	102.7	92.8

**Table 2:** Activation (ΔG^≠^) and reaction (ΔG) Gibbs free energies and rate constants (*k*) calculated at the M06-2X/6-311 + G(d,p) level of theory at 298.15 K in the gas phase.

Reactions	ΔG (kcal/mol)	ΔG^≠^ (kcal/mol)	*k* (M^−1^ s^−1^)
Calycosin-3′-OH + ^•^OH	−30.3	7.2	4.55 × 10^9^
Calycosin-7-OH + ^•^OH	−8.9	7.4	2.03 × 10^9^
Calycosin-3′-OH + ^•^OCH_3_	−14.8	12.1	6.72 × 10^5^
Calycosin-7-OH + ^•^OCH_3_	6.5	11.7	/

## Data Availability

Not applicable.

## Supplementary Materials

The following supporting information can be downloaded at: https://www.mdpi.com/article/10.3390/molecules28135039/s1. Figure S1: Optimized structures of the formononetin, calycosin, and calycosin-7-glucoside at the M06-2X/6-311 + G(d,p) level in the gas phase. Table S1: Calculated natural bond orbital (NBO) charges on the hydrogen atoms of phenolic hydroxyls for formononetin, calycosin, and calycosin-7-glucoside. Table S2: Optimized coordinates (x, y, z) of formononetin, calycosin, and calycosin-7-glucoside at the M06-2X level.

## Sample Availability

Samples of the compounds are not available from the authors.

## References

[1] Rahal A., Kumar A., Singh V., Yadav B., Tiwari R., Chakraborty S., Dhama K. (2014). Oxidative stress, prooxidants, and antioxidants: The interplay. Biomed. Res. Int..

[2] Pisoschi A.M., Pop A. (2015). The role of antioxidants in the chemistry of oxidative stress: A review. Eur. J. Med. Chem..

[3] Phaniendra A., Jestadi D.B., Periyasamy L. (2015). Free radicals: Properties, sources, targets, and their implication in various diseases. Indian J. Clin. Biochem..

[4] Hussain S.P., Hofseth L.J., Harris C.C. (2003). Radical causes of cancer. Nat. Rev. Cancer.

[5] Valko M., Leibfritz D., Moncol J., Cronin M.T., Mazur M., Telser J. (2007). Free radicals and antioxidants in normal physiological functions and human disease. Int. J. Biochem. Cell Biol..

[6] Gao H., Yao X.-S. (2019). Strengthen the research on the medicinal and edible substances to advance the development of the comprehensive healthcare industry of TCMs. Chin. J. Nat. Med..

[7] National Commission of Chinese Pharmacopoeia (2010). Pharmacopoeia of the People’s Republic of China.

[8] Li S.S., Sun Y., Huang J., Wang B., Gong Y.N., Fang Y.X., Liu Y.Y., Wang S.J., Guo Y., Wang H. (2020). Anti-tumor effects and mechanisms of Astragalus membranaceus (AM) and its specific immunopotentiation: Status and prospect. J. Ethnopharmacol..

[9] Chan J.Y., Koon J.C., Leung P.-C., Che C.-T., Fung K.-P. (2011). Suppression of lowdensity lipoprotein oxidation, vascular smooth muscle cell proliferation and migration by a herbal extract of Radix Astragali, Radix Codonopsis and Cortex Lycii. BMC Complement. Altern. Med..

[10] Ma C., Zhang J., Yang S., Hua Y., Su J., Shang Y., Wang Z., Feng K., Zhang J., Yang X. (2020). Astragalus flavone ameliorates atherosclerosis and hepatic steatosis Via inhibiting lipid-disorder and inflammation in apoE^−/−^ mice. Front. Pharm..

[11] Jalsrai A., Grecksch G., Becker A. (2010). Evaluation of the effects of Astragalus mongholicus Bunge saponin extract on central nervous system functions. J. Ethnopharmacol..

[12] Liu L.J., Li H.F., Xu F., Wang H.Y., Zhang Y.F., Liu G.X., Shang M.Y., Wang X., Cai S.Q. (2020). Exploring the in vivo existence forms (23 original constituents and 147 metabolites) of astragali radix total flavonoids and their distributions in rats using HPLC-DAD-ESI-IT-TOF-MSn. Molecules.

[13] Sheng Z., Jiang Y., Liu J., Yang B. (2021). UHPLC–MS/MS analysis on flavonoids composition in astragalus membranaceus and their antioxidant activity. Antioxidants.

[14] Bao X.-F., Cao P.-H., Zeng J., Xiao L.-M., Luo Z.-H., Zou J., Wang C.-X., Zhao Z.-X., Zhou Z.-Q., Zhi H. (2022). Bioactive pterocarpans from the root of *Astragalus membranaceus* var. mongholicus. Phytochemistry.

[15] Bratkov V.M., Shkondrov A.M., Zdraveva P.K., Krasteva I.N. (2016). Flavonoids from the genus Astragalus: Phytochemistry and biological activity. Pharm. Rev..

[16] Thaipong K., Boonprakob U., Crosby K., Cisneros Zevallos L., Byrne D.H. (2006). Comparison of ABTS, DPPH, FRAP, and ORAC assays for estimating antioxidant activity from guava fruit extracts. J. Food Compost. Anal..

[17] Mensor L.L., Menezes F.S., Leitão G.G., Reis A.S., Santos T.C.D., Coube C.S., Leitão S.G. (2001). Screening of Brazilian plant extracts for antioxidant activity by the use of DPPH free radical method. Phytother. Res..

[18] Sun T., Ho C.-T. (2005). Antioxidant activities of buckwheat extracts. Food Chem..

[19] Xu H., Guan D., Ma L. (2023). The bio-inspired heterogeneous single-cluster catalyst Ni100–Fe_4_S_4_ for enhanced electrochemical CO_2_ reduction to CH_4_. Nanoscale.

[20] Xiao W., Kiran G.K., Yoo K., Kim J.-H., Xu H. (2023). The dual-site adsorption and high redox activity enabled by hybrid organic-inorganic vanadyl ethylene glycolate for high-rate and long-durability lithium–sulfur batteries. Small.

[21] Lv Z., Xu H., Xu W., Peng B., Zhao C., Xie M., Lv X., Gao Y., Hu K., Fang Y. (2023). Quasi-topological intercalation mechanism of Bi_0.67_NbS_2_ enabling 100 C fast-charging for sodium-ion batteries. Adv. Energy Mater..

[22] Lu X.-Q., Chen Q., Tian X.-X., Mu Y.-W., Lu H.-G., Li S.-D. (2019). Predicting lanthanide boride inverse sandwich tubular molecular rotors with the smallest core–shell structure. Nanoscale.

[23] Wang G., Liu Y., Zhang L., An L., Chen R., Liu Y., Luo Q., Li Y., Wang H., Xue Y. (2020). Computational study on the antioxidant property of coumarin-fused coumarins. Food Chem..

[24] Xue Y., Teng Y., Chen M., Li Z., Wang G. (2021). Antioxidant activity and mechanism of avenanthramides: Double H^+^/e^−^ processes and role of the catechol, guaiacyl, and carboxyl groups. J. Agric. Food Chem..

[25] Purushothaman A., Teena Rose K.S., Jacob J.M., Varatharaj R., Shashikala K., Janardanan D. (2022). Curcumin analogues with improved antioxidant properties: A theoretical exploration. Food Chem..

[26] Amic A., Markovic Z., Markovic J.M.D., Milenkovic D., Stepanic V. (2020). Antioxidative potential of ferulic acid phenoxyl radical. Phytochemistry.

[27] Zheng Y.-Z., Zhou Y., Guo R., Fu Z.-M., Chen D.-F. (2020). Structure-antioxidant activity relationship of ferulic acid derivatives: Effect of ester groups at the end of the carbon side chain. LWT-Food Sci. Technol..

[28] Shang Y., Li X., Li Z., Zhou J., Qu L., Chen K. (2022). Theoretical study on the radical scavenging activity and mechanism of four kinds of Gnetin molecule. Food Chem..

[29] Yang L., Liu H., Xia D., Wang S. (2020). Antioxidant properties of camphene-based thiosemicarbazones: Experimental and theoretical evaluation. Molecules.

[30] Leopoldini M., Russo N., Toscano M. (2011). The molecular basis of working mechanism of natural polyphenolic antioxidants. Food Chem..

[31] Stepanić V., Trošelj K.G., Lučić B., Marković Z., Amić D. (2013). Bond dissociation free energy as a general parameter for flavonoid radical scavenging activity. Food Chem..

[32] Wright J.S., Johnson E.R., DiLabio G.A. (2001). Predicting the activity of phenolic antioxidants: Theoretical method, analysis of substituent effects, and application to major families of antioxidants. J. Am. Chem. Soc..

[33] Amića A., Markovićb Z., Kleinc E., Dimitrić Markovićd J.M., Milenković D. (2018). Theoretical study of the thermodynamics of the mechanisms underlying antiradical activity of cinnamic acid derivatives. Food Chem..

[34] Wang L., Yang F., Zhao X., Li Y. (2019). Effects of nitro- and amino-group on the antioxidant activity of genistein: A theoretical study. Food Chem..

[35] Zheng Y.-Z., Deng G., Liang Q., Chen D.-F., Guo R., Lai R.-C. (2017). Antioxidant activity of quercetin and its glucosides from propolis: A theoretical study. Sci. Rep..

[36] Lu T. (2022). Molclus Program. Beijing Kein Research Center for Natural Science. Version 1.9.9.9. https://www.keinsci.com/research/molclus.html.

[37] Zhao Y., Truhlar D.G. (2008). The M06 suite of density functionals for main group thermochemistry, thermochemical kinetics, noncovalent interactions, excited states, and transition elements: Two new functionals and systematic testing of four M06-class functionals and 12 other functionals. Theor. Chem. Acc..

[38] Krishnan R., Binkley J.S., Seeger R., Pople J.A. (1980). Self-consistent molecular orbital methods. XX. A basis set for correlated wave functions. J. Chem. Phys..

[39] Frisch M.J., Trucks G.W., Schlegel H.B., Scuseria G.E., Robb M., Cheeseman J.R., Scalmani G., Barone V.P.G.A., Petersson G.A., Nakatsuji H.J.R.A. (2016). Gaussian 16, Revision A.03.

[40] Marenich A.V., Cramer C.J., Truhlar D.G. (2009). Universal solvation model based on solute electron density and on a continuum model of the solvent defined by the bulk dielectric constant and atomic surface tensions. J. Phys. Chem. B.

[41] Glendening P.E.D., Badenhoop J.K., Reed A.E., Carpenter J.E., Bohmann J.A., Morales C.M., Landis C.R., Weinhold F. NBO 6.0. https://nbo6.chem.wisc.edu/.

[42] Zheng Y.-Z., Fu Z.-M., Guo R., Chen D.-F., Zhang Y.-C. (2021). The important role of benzylic C–H bond in the antioxidant behaviours of the xanthones. J. Food Compos. Anal..

[43] Xue Y., Liu Y., Luo Q., Wang H., Chen R., Liu Y., Li Y. (2018). Antiradical activity and mechanism of coumarin−chalcone hybrids: Theoretical insights. J. Phys. Chem. A.

[44] Canneaux S., Bohr F., Henon E. (2014). KiSThelP: A program to predict thermodynamic properties and rate constants from quantum chemistry results. J. Comput. Chem..

[45] Vo V.Q., Bay M.V., Nam P.C., Mechler A. (2019). Is indolinonic hydroxylamine a promising artificial antioxidant?. J. Phys. Chem. B.

[46] Dimić D., Milenković D., Marković J.D., Marković Z. (2017). Antiradical activity of catecholamines and metabolites of dopamine: Theoretical and experimental study. Phys. Chem. Chem. Phys..

[47] Garzón A., Bravo I., Barbero A.J., Albaladejo J. (2014). Mechanistic and kinetic study on the reactions of coumaric acids with reactive oxygen species: A DFT approach. J. Agric. Food Chem..

[48] Galano A., Mazzone G., Alvarez-Diduk R., Marino T., Alvarez-Idaboy J.R., Russo N. (2016). Food antioxidants: Chemical insights at the molecular level. Annu. Rev. Food Sci. T..

[49] Fukui K. (1981). The path of chemical reactions-the IRC approach. Acc. Chem. Res..

[50] Bartmess J.E. (1994). Thermodynamics of the electron and the proton. J. Phys. Chem..

[51] Rimarcík J., Lukes V., Klein E., Ilcin M. (2010). Study of the solvent effect on the enthalpies of homolytic and heterolytic N–H bond cleavage in p-phenylenediamine and tetracyano-p-phenylenediamine. J. Mol. Struc. Theor. Chem..

[52] Markovic Z., Tosovic J., Milenkovic D., Markovic S. (2016). Revisiting the solvation enthalpies and free energies of the proton and electron in various solvents. Comput. Theor. Chem..

[53] Eyring H. (1935). The activated complex in chemical reactions. J. Chem. Phys..

[54] Wigner E. (1932). On the quantum correction for thermodynamic equilibrium. Phys. Rev..

