# Supplementary material for: Radical Scavenging Capability and Mechanism of Three Isoflavonoids Extracted from Radix Astragali: A Theoretical Study
Source: Molecules. 2023 Jun 28;28(13):5039. doi: 10.3390/molecules28135039 (PMC10343223; doi:10.3390/molecules28135039)
Supplement: Supplementary file 1 [file molecules-28-05039-s001.zip › molecules-2466481-supplementary.pdf]

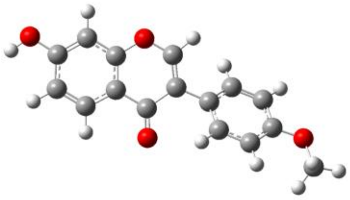

Formononetin

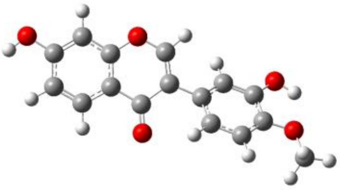

Calycosin

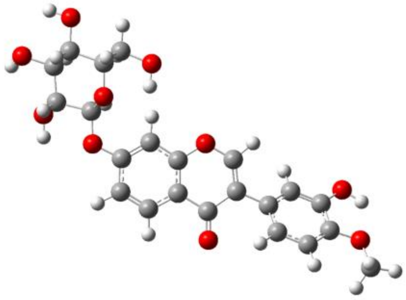

Calycosin-7-glucoside

Figure S1 Optimized structures of the formononetin, calycosin, and calycosin-7-glucoside at M06-2X/6-311+G(d,p) level in the gas phase.

Table S1 Calculated natural bond orbital (NBO) charges on the hydrogen atoms of phenolic hydroxyls for formononetin, calycosin, and calycosin-7-glucoside.

Reactions	Formononetin	Calycosin	Calycosin-7-glucoside
3′-OH		0.494	0.494
7-OH	0.478	0.478	

Table S2 Optimized coordinates (x, y, z) of formononetin, calycosin, and calycosin-7-glucoside at M06-2X level.

Formononetin

O	1.84472700	-1.77071800	0.39298200
O	0.31503100	1.93376200	-0.35323400
O	-5.71419200	-0.24474600	-0.21185600
O	6.26778000	-0.28085900	0.08010600
C	-0.08269000	-0.37411200	0.06341900
C	2.20962700	0.55230500	-0.08927900
C	-1.55722300	-0.29682800	-0.01764500
C	0.76075900	0.82085100	-0.14690000
C	2.69143200	-0.72953400	0.17416100
C	0.51626200	-1.55041000	0.33363200
C	3.14154400	1.57601400	-0.30170600
C	4.05225800	-1.01163800	0.23314100
C	-2.25634700	0.76803400	0.54756700
C	-2.29164000	-1.30662600	-0.65359500
C	4.94822400	0.02095200	0.01831100
C	4.49445000	1.32368700	-0.25148500
C	-3.64665600	0.82090200	0.50680900
C	-3.67307100	-1.26835100	-0.69881400
C	-4.36082300	-0.20259400	-0.11446400
C	-6.44688600	0.82749800	0.34303200
H	-0.03400400	-2.45908900	0.54211500
H	2.75966300	2.56898400	-0.50692200
H	-1.71021900	1.57256000	1.02182200
H	-1.76882600	-2.12188000	-1.14205300
H	4.40031900	-2.01436400	0.44165500
H	5.21198400	2.12022100	-0.41772700
H	-4.15346500	1.66289300	0.95808800
H	-4.24456600	-2.04217800	-1.19604300
H	6.79424200	0.50662800	-0.08272400
H	-6.28019000	0.90381600	1.42197400
H	-7.49469700	0.60627000	0.15365100
H	-6.18233500	1.77572500	-0.13493000

Calycosin

O	2.01938500	1.67375100	-0.59476000
O	0.64588800	-2.00279700	0.50891300
O	-5.47120500	-0.07057300	0.03193200
O	-4.21958100	2.08373400	0.90988700
O	6.49822800	0.43495900	-0.05815000
C	0.15113300	0.22956600	-0.15142300
C	2.47915900	-0.56677500	0.13265200
C	-1.32151400	0.09426600	-0.09291000
C	1.04279300	-0.89676700	0.19481600
C	2.90795700	0.70193700	-0.25548200
C	0.70191700	1.39915500	-0.53159600
C	-2.10009100	1.14733500	0.40517200
C	-1.95469700	-1.05921700	-0.54856400
C	3.45185700	-1.51766100	0.46597400
C	4.25543500	1.04061800	-0.32210300
C	-3.47888300	1.05730800	0.42167000
C	-4.10830100	-0.10375200	-0.05010100
C	-3.34517400	-1.15756300	-0.52946500
C	5.19262900	0.07934000	0.01373200
C	4.79275900	-1.20820600	0.41123100
C	-6.17860300	-1.22050600	-0.38854100
H	0.11517200	2.25519800	-0.83960700
H	-1.64300200	2.04104000	0.81395900
H	-1.36557800	-1.88935900	-0.91167900
H	3.11120000	-2.50077400	0.76822300
H	4.56166800	2.03175800	-0.62857500
H	-3.81879400	-2.06188200	-0.88802600
H	5.54199500	-1.94824400	0.67192100
H	-5.14877700	1.82919300	0.86189400
H	7.05603400	-0.30578700	0.19471700
H	-5.99623000	-1.42358500	-1.44772500
H	-7.23288500	-1.00267300	-0.23546200
H	-5.89278700	-2.09194600	0.20741600

Calycosin-7-glucoside

C	-4.76877000	-1.57273200	-0.34509700
C	-6.23566100	-1.20060300	-0.15708200
C	-6.34650000	-0.07383400	0.85758200
C	-5.46822600	1.09724600	0.45259100
C	-4.04802100	0.59044200	0.24525200
H	-6.63708000	-0.84392800	-1.11678800
H	-4.36390900	-1.95825600	0.60244800
H	-5.82186900	1.51303800	-0.49941600
H	-3.63770800	0.20353800	1.19217200
H	-6.00006100	-0.44753800	1.83284100
O	0.74906900	-0.82713000	0.22862500
O	2.99773700	2.46654700	-0.60679600
O	8.46479100	-0.81168000	0.26663800
O	6.82843100	-1.87309000	2.04788500
O	-3.28350800	1.66887400	-0.19922300
C	2.92426600	0.15993400	-0.02283600
C	0.86463200	1.50810000	-0.30302600
C	4.38935900	-0.04141600	0.03412200
C	2.34008200	1.47935800	-0.33967500
C	0.13573900	0.35996200	-0.01745100
C	2.09570300	-0.87666600	0.21192000
C	4.94430900	-0.85104700	1.03393300
C	5.23063900	0.54315100	-0.90897900
C	0.15686600	2.69321200	-0.55774000
C	-1.26078500	0.34852800	0.01998200
C	6.30472200	-1.08973500	1.07220900
C	7.14302400	-0.50633200	0.11086100
C	6.60451700	0.30954500	-0.87234900
C	-1.92364200	1.53810200	-0.22521700
C	-1.21627100	2.71639400	-0.52025200
C	9.37999000	-0.23585900	-0.64482500
H	2.44641500	-1.88171300	0.40849900
H	4.32884800	-1.28446800	1.81374400
H	4.81936700	1.18993900	-1.67090700
H	0.73177500	3.58412100	-0.78021900
H	-1.77010300	-0.58637700	0.21015800
H	7.24216600	0.77184600	-1.61418600

H	-1.77772100	3.62172800	-0.71154800
H	7.78468200	-1.90315900	1.92432300
H	9.16493300	-0.55846600	-1.66754700
H	10.36556200	-0.58878000	-0.35084400
H	9.34863100	0.85599400	-0.58924000
O	-4.03770600	-0.41078600	-0.73811400
O	-5.54298400	2.04908800	1.48989700
H	-5.03365300	2.82423200	1.23403200
O	-7.70750400	0.29114500	0.93465500
H	-7.78684000	1.02301100	1.55541000
O	-6.92552800	-2.34744600	0.28003900
H	-7.81461000	-2.07312800	0.52996100
C	-4.54493200	-2.60139900	-1.43557900
H	-5.04466800	-3.52978300	-1.16143500
H	-4.98202400	-2.22870700	-2.37077500
O	-3.17191700	-2.88184500	-1.58758500
H	-2.73541000	-2.05866200	-1.82889000
